# Induction of autophagy-dependent ferroptosis to eliminate drug-tolerant human retinoblastoma cells

**DOI:** 10.1038/s41419-022-04974-8

**Published:** 2022-06-02

**Authors:** Ke Liu, Jun Huang, Jiao Liu, Daniel J. Klionsky, Rui Kang, Daolin Tang

**Affiliations:** 1 Department of Ophthalmology, The Second Xiangya Hospital, Central South University, Changsha, China; 2 Department of Orthopaedics, The Second Xiangya Hospital, Central South University, Changsha, China; 3 DAMP Laboratory, The Third Affiliated Hospital, Guangzhou Medical University, Guangzhou, China; 4 Life Sciences Institute and Department of Molecular, Cellular and Developmental Biology, University of Michigan, Ann Arbor, MI 48109 USA; 5 Department of Surgery, UT Southwestern Medical Center, Dallas, TX USA

**Keywords:** Eye cancer, Macroautophagy

## Abstract

Carboplatin is the most used first-line drug for the treatment of human retinoblastoma (RB), a rare form of cancer in infancy and childhood. However, the clinical application of carboplatin is restricted due to the emergence of acquired multi-drug resistance (MDR) after long-term treatment. Here, we report a new strategy to eliminate MDR RB cells by inducing autophagy-dependent ferroptosis. Compared with parent cells, carboplatin-resistant human RB cells have higher autophagy activity, which drives the formation of MDR to other chemotherapeutic drugs (e.g., etoposide and vincristine). In addition to confirming the traditional strategy of inhibiting autophagy to overcome MDR, we also establish an approach of inducing selective ferritinophagy to eliminate drug-resistant cells. We evaluate the effectiveness and safety of 4-octyl itaconate, a cell-permeable derivative of the metabolite itaconate, in inducing ferritinophagy-dependent ferroptosis in the treatment of MDR RB cells in vitro and in xenograft mouse models. These findings may provide essential clues for initiating clinical trials that target autophagy-dependent ferroptosis to kill drug-tolerant persistent cells during RB therapy.

## Introduction

The rare cancer retinoblastoma (RB) is an aggressive and the most common intraocular cancer in children, mainly caused by mutations in the tumor suppressor gene *RB1* (RB transcriptional corepressor 1) [1]. This cancer usually occurs before the age of five, and most often occurs in children under two years of age [2]. This disease accounts for 3% of childhood cancers, and the global survival rate of RB patients is less than 30% [3]. Although treatment options and recommendations depend on the type and stage of RB, the most-used method is chemotherapy, including systemic, subconjunctival, intra-arterial, and intravitreal routes [4]. Among the first-line chemotherapy drugs for RB, carboplatin is an alkylating agent that induces apoptosis by interfering with DNA repair [5–7]. However, patients with long-term treatment of carboplatin often develop drug resistance by different mechanisms [8–10]. Thus, there is an urgent need to identify new targets to improve the therapeutic effect and overcome the resistance of RB therapy.

Macroautophagy (hereafter autophagy) is a lysosome-mediated degradation pathway [11], which affects all stages of tumor initiation and development [12–14]. Autophagy is a defense mechanism that promotes survival by eliminating damaged organelles and protein aggregates [11]. However, unrestricted activation of autophagy may lead to cell death, termed autophagy-dependent cell death [15]. We and others have previously demonstrated that upregulation of autophagy contributes to the therapy resistance of RB cells [16–18]. Because autophagy plays a fundamental role in the control of homeostasis in normal tissues [19, 20], the strategy of inhibiting autophagy to restore drug sensitivity may cause side effects and even toxicity [21]. Alternatively, inducing autophagy-dependent cell death is becoming a promising strategy for tumor therapy is some solid cancers [22–24]. However, this idea has not been tested in rare cancer treatment.

In this study, we provide the evidence that inducing autophagy-dependent ferroptosis, which is a type of oxidative cell death driven by lipid peroxidation [25], is an effective strategy to eliminate drug-tolerant RB cells. Especially, we demonstrate that the anticancer potential of an itaconate derivative in RB cell relies on ferritinophagy-mediated ferroptosis in cell cultures and mouse models, highlighting a new metabolite strategy for rare cancer therapy.

## Results

### Autophagy promotes multidrug resistance in RB cells

**Fig. 1:**
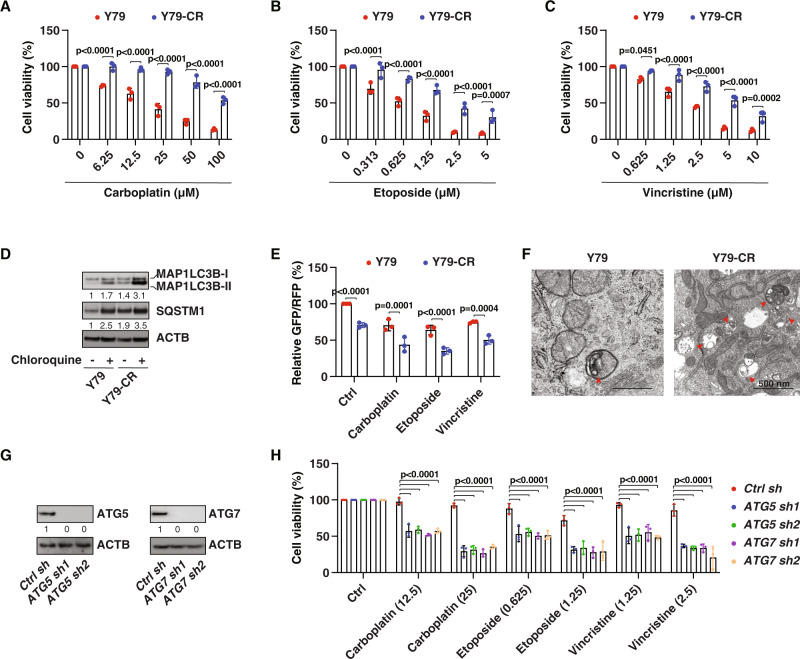
The upregulation of autophagy promotes multi-drug resistance. **A**–**C** Y79 and Y79-CR cells were treated with the indicated chemotherapy drugs for 24 h, and cell viability was assayed (two-way ANOVA with Tukey’s multiple comparisons test; data are presented as mean ± SD; *n* = 3 biologically independent samples). **D** Western blot analysis of protein expression in Y79 and Y79-CR cells in the absence or presence of chloroquine (50 µM) treatment for 6 h. **E** The GFP-LC3-RFP-LC3ΔG probe was used to measure autophagic flux in Y79 and Y79-CR cells following treatment with carboplatin (25 µM), etoposide (1.25 µM), or vincristine (2.5 µM) for 6 h (two-way ANOVA with Tukey’s multiple comparisons test; data are presented as mean ± SD of relative GFP:RFP ratio; *n* = 3 biologically independent samples). **F** The representative transmission electron microscopy image of autophagic vacuoles (red arrowheads) in Y79 and Y79-CR cells. Bar: 500 nm. **G** Western blot analysis of protein expression in *ATG5*- or *ATG7*-knockdown Y79-CR cells. **H** Cell viability analysis of the indicated Y79-CR cells following treatment with carboplatin (12.5 and 25 µM), etoposide (0.625 and 1.25 µM), or vincristine (1.25 and 2.5 µM) for 24 h (two-way ANOVA with Tukey’s multiple comparisons test; data are presented as mean ± SD; *n* = 3 biologically independent samples).

Y79 cells are the oldest and most commonly used human RB cell lines to study treatment response [26]. To study the mechanism of carboplatin resistance in RB cells, we established a carboplatin-resistant cell line, termed Y79-CR cells, by exposing Y79 cells to carboplatin through the limiting drug dilution method [27]. Compared with the parent Y79, Y79-CR cells were resistant to carboplatin-induced growth inhibition (Fig. 1A). To determine whether Y79-CR cells are also resistant to other anti-tumor reagents, we treated cells with etoposide and vincristine, which are widely used chemotherapy drugs in RB therapy [4]. Surprisingly, Y79-CR cells were also resistant to etoposide (Fig. 1B) and vincristine (Fig. 1C), indicating that Y79-CR cells have a multi-drug resistance (MDR) mechanism.

Because autophagy is one of the main mechanisms of acquired MDR in various cancers [28], we measured the level of autophagy in parent and drug-tolerant RB cells. Compared with parent cells, the protein level of autophagosome marker MAP1LC3B (microtubule associated protein 1 light chain 3 beta)-II was upregulated, while the protein level of the autophagy receptor and substrate SQSTM1 (sequestosome 1) was also upregulated in Y79-CR cells (Fig. 1D). Importantly, additional treatment with chloroquine, a potent inhibitor of autophagy that blocks the fusion of autophagosomes with lysosomes and lowers lysosomal hydrolytic activity by altering the acidic environment of lysosomes [29], further increased the level of MAP1LC3B-II and SQSTM1 in Y79 and Y79-CR cells, especially Y79-CR cells (Fig. 1D); this latter result indicates that the Y79-CR cells without treatment displayed an increase in autophagy as opposed to a block in lysosome-dependent turnover. The GFP-LC3-RFP-LC3ΔG construct is an autophagy flux probe [30]. The decrease in GFP fluorescence relative to RFP fluorescence indicates an increase in autophagy flux because the GFP-LC3 delivered to the lysosome is quenched, whereas RFP-LC3∆G remains in the cytosol [30]. Analysis of autophagic flux by estimating the GFP: RFP ratio also confirmed that Y79-CR cells had increased autophagic flux activity in the absence or presence of carboplatin, etoposide, or vincristine (Fig. 1E). Moreover, transmission electron microscopy, an indispensable standard method to monitor autophagy [29], showed an increase in autophagic vacuoles in Y79-CR cells compared to Y79 cells (Fig. 1F).

Next, we determined whether inhibition of autophagy can restore the response of Y79-CR cells to chemotherapeutic drugs. We used shRNA-mediated RNAi to inhibit the expression of two core autophagy regulators, namely ATG5 (autophagy-related 5) and ATG7, in Y79-CR cells. Western blotting confirmed that the RNAi efficiency reached more than 95% inhibition of the expression of ATG5 or ATG7 (Fig. 1G). In the *ATG5*- or *ATG7*-knockdown cells, the anticancer activity of carboplatin, etoposide, and vincristine was restored (Fig. 1H). These findings support the hypothesis that upregulated autophagy leads to the formation of MDR.

### Induction of ferroptosis eliminates drug-resistant RB cells

**Fig. 2:**
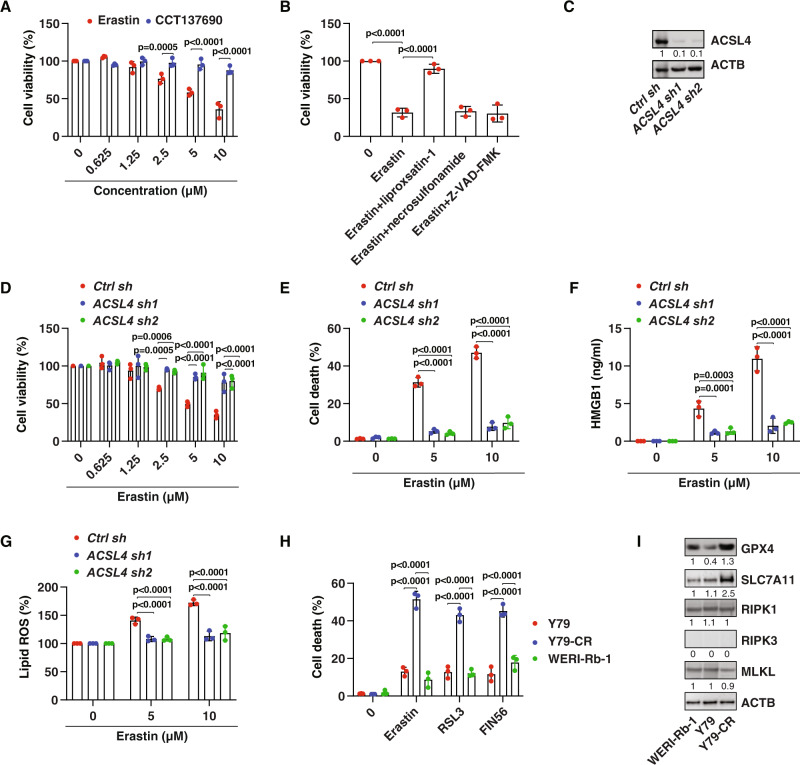
Induction of ferroptosis eliminates drug-resistant cells. **A** Y79-CR cells were treated with erastin (0.625–10 µM) or CCT137690 (0.625–10 µM) for 24 h, and cell viability was assayed (two-way ANOVA with Tukey’s multiple comparisons test; data are presented as mean ± SD; *n* = 3 biologically independent samples). **B** Y79-CR cells were treated with erastin (10 µM) in the absence or presence of liproxsatin-1 (1 µM), necrosulfonamide (1 µM), or Z-VAD-FMK (10 µM) for 24 h, and cell viability was assayed (two-way ANOVA with Tukey’s multiple comparisons test; data are presented as mean ± SD; *n* = 3 biologically independent samples). **C** Western blot analysis of protein expression in control and *ACSL4*-knockdown Y79-CR cells. **D** Cell viability analysis of the indicated Y79-CR cells following treatment with erastin (0.625–10 µM) for 24 h (two-way ANOVA with Tukey’s multiple comparisons test; data are presented as mean ± SD; *n* = 3 biologically independent samples). **E**–**G** The indicated Y79-CR cells were treated with erastin (5–10 µM) for 24 h, and then cell death (**E**), HMGB1 release (**F**), and lipid ROS (**G**) were assayed (two-way ANOVA with Tukey’s multiple comparisons test; data are presented as mean ± SD; *n* = 3 biologically independent samples). **H** The indicated human RB cells were treated with erastin (10 µM), RSL3 (1 µM), or FIN56 (1 µM) for 24 h, and cell death was assayed (two-way ANOVA with Tukey’s multiple comparisons test; data are presented as mean ± SD; *n* = 3 biologically independent samples). **I** Western blot analysis of protein expression in the indicated human RB cell lines.

Since the anticancer activity of carboplatin mainly acts by initiating apoptosis [31], we next examined whether induction of non-apoptotic cell death can eliminate drug-resistant cells. We focused on two extensively studied non-apoptotic cell death modalities in cancer therapy: necroptosis [32] and ferroptosis [33]. We treated Y79-CR cells with CCT137690 [34] or erastin [35], which are small molecule inducers of necroptosis and ferroptosis, respectively. Unlike CCT137690, erastin dose-dependently caused growth inhibition in Y79-CR cells (Fig. 2A). This tumor-suppressive effect of erastin on Y79-CR cells was reversed by addition of the ferroptosis inhibitor liproxsatin-1, but not the necroptosis inhibitor necrosulfonamide or the apoptosis inhibitor Z-VAD-FMK (Fig. 2B). In line with the drug and chemical study, the suppression of ACSL4 (acyl-CoA synthetase long chain family member 4), a key promoter of various types of ferroptosis [36–38], blocked erastin-induced growth inhibition in Y79-CR cells (Fig. 2C, D). Subsequent analysis of cell death, danger/damage-associated molecular patterns (DAMPs, such as HMGB1 [high mobility group box 1] [39]), and lipid peroxidation using BODIPY 581/591 C11 indicator confirmed that erastin induced ACSL4-dependent ferroptosis in Y79-CR cells (Fig. 2E–G). Other classical ferroptosis inducers, including RSL3 [40] and FIN56 [41], also suppressed tumor growth in Y79-CR cells (Fig. 2H). However, compared to Y79-CR cells, the anticancer activity of ferroptosis inducers (erastin, RSL3, and FIN56) was reduced in the parent Y79 cells and in another well-characterized human RB cell line, WERI-Rb-1 (Fig. 2H), highlighting a relatively selective role in the induction of ferroptosis to eliminate drug-resistant RB cells.

To determine the mechanism by which carboplatin-resistant RB cells are sensitive to ferroptosis activators, we examined the expression of SLC7A11 (solute carrier family 7 member 11) and GPX4 (glutathione peroxidase 4), which are direct drug targets for the induction of ferroptosis by erastin, RSL3, or FIN56 [35, 40–42]. Western blot analysis revealed that the protein expression of SLC7A11 and GPX4 was upregulated in Y79-CR cells compared with Y79 and WERI-Rb-1 cells (Fig. 2I). In contrast, the protein expression of necroptosis regulators, RIPK1 (receptor-interacting serine/threonine kinase 1) and MLKL (mixed lineage kinase domain-like pseudokinase) [32], did not differ between Y79-CR, Y79, and WERI-Rb-1 cells (Fig. 2I). In addition, the protein expression of another necroptosis mediator, RIPK3 (receptor-interacting serine/threonine kinase 3) [32], was not detected in Y79-CR, Y79, and WERI-Rb-1 cells (Fig. 2I). These cell death mediator assays may partially explain why carboplatin-resistant human RB cells are sensitive to targeted therapy with ferroptosis inducers, rather than necroptosis inducers.

### Ferritinophagy mediates ferroptosis in drug-resistant RB cells

**Fig. 3:**
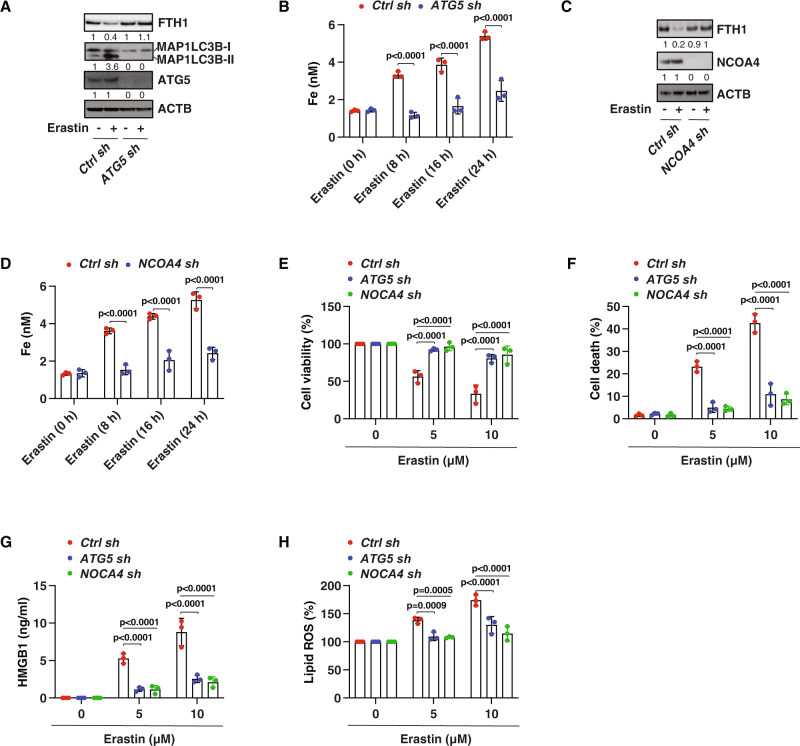
Ferritinophagy mediates ferroptotic tumor suppression. **A** Western blot analysis of protein expression in control and *ATG5*-knockdown Y79-CR cells following treatment with erastin (10 µM) for 24 h. **B** Analysis of intracellular free iron in control and *ATG5*-knockdown Y79-CR cells following treatment with erastin (10 µM) for 8–24 h (two-way ANOVA with Tukey’s multiple comparisons test; data are presented as mean ± SD; *n* = 3 biologically independent samples). **C** Western blot analysis of protein expression in control and *NCOA4*-knockdown Y79-CR cells following treatment with erastin (10 µM) for 24 h. **D** Analysis of intracellular free iron in control and *NCOA4*-knockdown Y79-CR cells following treatment with erastin (10 µM) for 8–24 h (two-way ANOVA with Tukey’s multiple comparisons test; data are presented as mean ± SD; *n* = 3 biologically independent samples). **E**–**H** Indicated Y79-CR cells were treated with erastin (5–10 µM) for 24 h, and then cell viability (**E**), cell death (**F**), HMGB1 release (**G**), and lipid ROS (**H**) were assayed (two-way ANOVA with Tukey’s multiple comparisons test; data are presented as mean ± SD; *n* = 3 biologically independent samples).

Accumulated evidence shows that ferroptosis is a type of autophagy-dependent cell death, which requires the degradation of anti-ferroptotic regulators by autophagy [43]. Ferritinophagy-mediated degradation of the iron storage protein ferritin increases the level of bioavailable ferrous iron in cells, leading to the production of reactive oxygen species (ROS) and subsequent lipid peroxidation through the Fenton reaction [44]. Given these findings previously established in non-RB cancer cells, we next asked whether ferritinophagy is involved in ferroptosis-mediated tumor suppression in drug-resistant RB cells. First, the knockdown of *ATG5* by shRNA inhibited erastin-induced protein degradation of FTH1 (ferritin heavy chain 1) as well as iron accumulation in Y79-CR cells (Fig. 3A, B). Second, genetic silencing of NCOA4 (nuclear receptor coactivator 4), a selective autophagy receptor responsible for ferritinophagy [45, 46], also blocked FTH1 degradation and subsequent iron accumulation in Y79-CR cells during ferroptosis (Fig. 3C, D). Third, cell viability, cell death, HMGB1 release, and lipid peroxidation analysis further confirmed the role of ATG5 and NCOA4 in mediating erastin-induced ferroptosis in *ATG5*-knockdown or *NCOA4*-knockdown Y79-CR cells (Fig. 3E–H). These findings support the conclusion that ferritinophagy is required for tumor suppression caused by ferroptosis activators in MDR RB cells.

### Induction of ferritinophagy eliminates drug-resistant RB cells

**Fig. 4:**
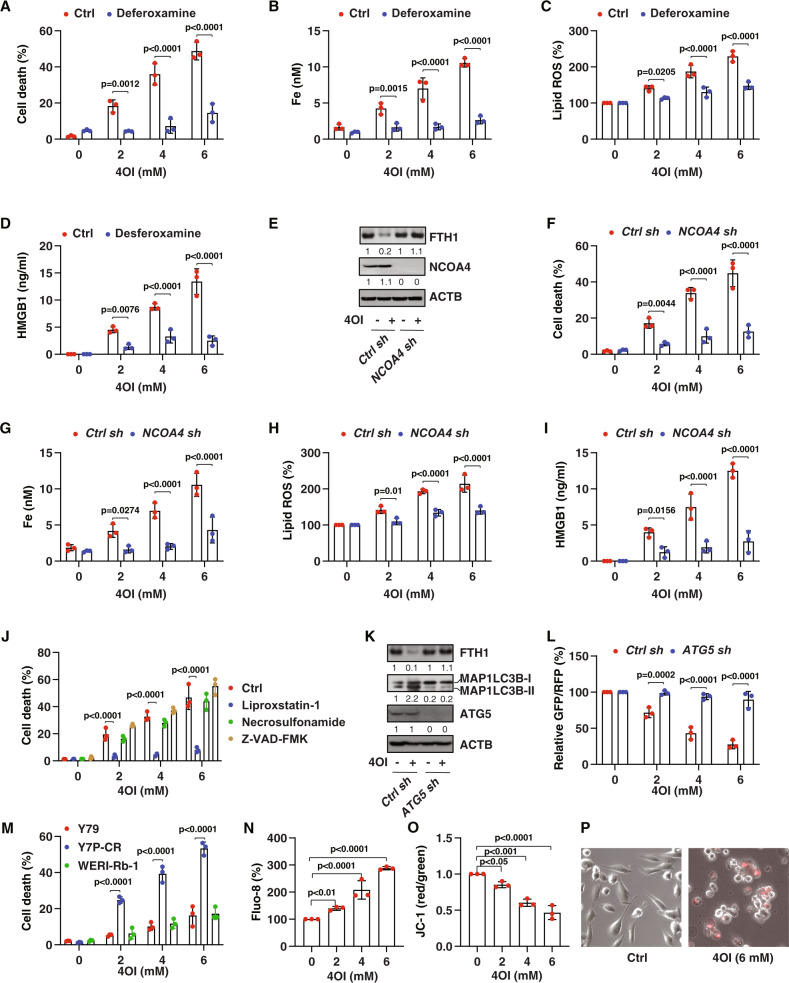
Itaconate-induced ferritinophagy eliminates drug-resistant cells. Y79-CR cells were treated with 4OI (2–6 mM) in the absence or presence of deferoxamine (50 µM) for 24 h, and then cell death (**A**), intracellular free iron (**B**), lipid ROS (**C**), and HMGB1 release (**D**) were assayed (two-way ANOVA with Tukey’s multiple comparisons test; data are presented as mean ± SD; *n* = 3 biologically independent samples). **E** Western blot analysis of protein expression in control and *NCOA4*-knockdown Y79-CR cells following treatment with 4OI (4 mM) for 24 h. The indicated Y79-CR cells were treated with 4OI (2–6 mM) for 24 h, and then cell death (**F**), intracellular free iron (**G**), lipid ROS (**H**), and HMGB1 release (**I**) were assayed (two-way ANOVA with Tukey’s multiple comparisons test; data are presented as mean ± SD; *n* = 3 biologically independent samples). **J** Y79-CR cells were treated with 4OI (2–6 mM) in the absence or presence of liproxsatin-1 (1 µM), necrosulfonamide (1 µM), or Z-VAD-FMK (10 µM) for 24 h, and then cell death was assayed (two-way ANOVA with Tukey’s multiple comparisons test; data are presented as mean ± SD; *n* = 3 biologically independent samples). **M** The indicated human RB cells were treated with 4OI (2–6 mM) for 24 h, and then cell death was assayed (two-way ANOVA with Tukey’s multiple comparisons test; data are presented as mean ± SD; *n* = 3 biologically independent samples). **N**–**P** Y79-CR cells were treated with indicated 4OI for 24 h, and then cytosolic calcium (Fluo-8 staining), mitochondrial membrane potential (JC-1 staining), and cell morphology (propidium iodide staining) were assayed (one-way ANOVA with Tukey’s multiple comparisons test; data are presented as mean ± SD; *n* = 3 biologically independent samples).

Although erastin, RSL3, or FIN56 have potential activity to induce ferroptosis in vitro, their metabolic instability may limit their application in vivo [33]. Alternatively, the induction of ferroptosis by metabolites is a potential strategy to suppress tumor growth in vitro and in vivo [47]. Itaconate, an anti-inflammatory metabolite of the tricarboxylic acid cycle, has recently been determined to induce ferritinophagy-dependent ferroptosis in leukemia and pancreatic cancer cells [48]. These emerging findings prompted us to examine the ability of itaconate to suppress the growth of drug-resistant RB cells. 4-octyl itaconate (4OI), the cellular permeable derivate of itaconate [49], was used to treat Y79-CR cells. We chose a 4OI concentration of 2–6 mM, because in inflammatory diseases the concentration of itaconate can rise to millimolar levels [50, 51]. 4OI dose-dependently induced cell death associated with increased intracellular free iron accumulation, lipid peroxidation, and HMGB1 release in Y79-CR cells (Fig. 4A–D). In contrast, the iron chelator deferoxamine reversed these effects induced by 4OI (Fig. 4A–D), meaning that iron is required for the anticancer activity of 4OI.

To determine whether ferritinophagy is required for 4OI-mediated tumor suppression, we assayed the level of FTH1 in control and *NCOA4*-knockdown Y79-CR cells (Fig. 4E). The knockdown of *NCOA4* prevented 4OI-induced FTH1 degradation, iron accumulation, lipid peroxidation, cell death, and subsequent HMGB1 release (Fig. 4E–I). Consistent with the protective effect seem with the suppression of NCOA4 (Fig. 3F), the ferroptosis inhibitor liproxstatin-1, but not other cell death inhibitors (necrosulfonamide and Z-VAD-FMK), blocked 4OI-induced cell death in Y79-CR cells (Fig. 4J). Analysis of MAP1LC3B-II expression by western blot and autophagic flux using the GFP-LC3-RFP-LC3ΔG probe also revealed that 4OI increased autophagy (i.e., increased MAP1LC3B-II and decreased the GFP:RFP ratio) in control, but not *ATG5*-knockdown, Y79-CR cells (Fig. 4K, L). Like erastin, 4OI had a higher anticancer activity in Y79-CR cells, compared to parent Y79 or WERI-Rb-1 cells (Fig. 4M), further supporting the idea that drug-resistant RB cells are particularly sensitive to the induction of ferritinophagy.

Previous studies have shown that ferroptosis is associated with increased calcium influx [52, 53], decreased mitochondrial membrane potential [35], and necrosis-like morphology [35]. As expected, 4OI increased cytosolic calcium (Fig. 4N) and decreased mitochondrial membrane potential (Fig. 4O) in Y79-CR cells with propidium iodide-positive and necrosis-like morphology (Fig. 4P).

### Evaluate effectiveness and safety of an itaconate derivative in RB therapy

**Fig. 5:**
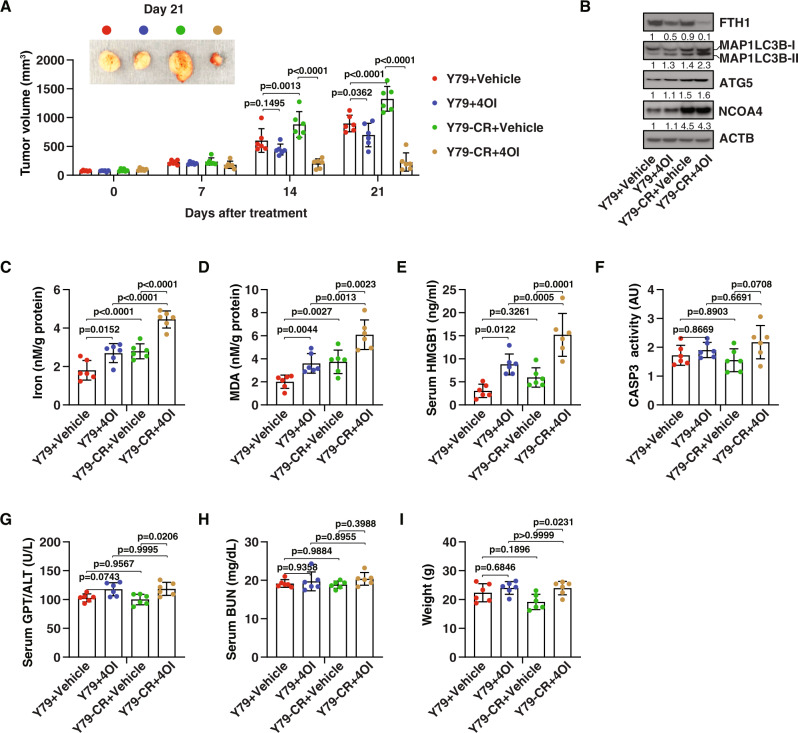
Itaconate suppresses tumor growth in vivo. **A** Athymic nude mice were injected subcutaneously with Y79 and Y79-CR cells for 7 days and then given intraperitoneal treatment with 4OI (50 mg/kg, once every other day) at day 7 for 2 weeks. Tumor volumes were calculated weekly (two-way ANOVA with Tukey’s multiple comparisons test; data are presented as mean ± SD; *n* = 6 mice/group). **B**–**I** The indicated markers in serum or tumors as well as body weight at day 21 were assayed (one-way ANOVA with Tukey’s multiple comparisons test; data are presented as mean ± SD; *n* = 6 mice/group).

A previous study reported that 50 mg/kg of 4OI is safe and effectively prevents the death of C57BL/6 mice induced by endotoxemia [49]. Next, we used a xenograft mouse model to examine the effect of 4OI at the same dose on tumor suppression of RB cells in vivo. Human Y79 and Y79-CR cells were implanted subcutaneously into the flank of immunodeficient nude mice. One week later, the tumor-bearing mice were injected with 4OI (50 mg/kg/day, once every other day, for 2 weeks). Consistent with our in vitro data (Fig. 4M), 4OI showed greater anticancer activity in Y79-CR cells than in Y79 cells (Fig. 5A). Subsequent analysis of MAP1LC3B-II protein expression (Fig. 5B), free iron levels (Fig. 5C), and malondialdehyde (MDA, one of the final products of lipid peroxidation [54]) (Fig. 5D) in tumors as well as serum HMGB1 (Fig. 5E) at day 21 after treatment suggested that 4OI-treated Y79-CR cells had higher levels of autophagic and ferroptotic responses. Accordingly, the protein expression of FTH1 was downregulated by 4OI (Fig. 5B), supporting the concept that 4OI can trigger ferritinophagy for the degradation of ferritin. In contrast, administration of 4OI in mice had no significant effect on the activity of CASP3 (caspase 3) in isolated tumor tissues (Fig. 5F), indicating that 4OI-mediated tumor suppression is independent of caspase-mediated apoptosis.

We also monitored liver and kidney functions by measuring the serum levels of GPT/ALT (glutamic--pyruvic transaminase) and blood urea nitrogen (BUN). Compared with the untreated group, serum GPT/ALT (but not serum BUN) mildly increased by about 5–10% after 4OI treatment (Fig. 5G, H), supporting the previous observation that liver is the primary site for itaconate metabolism [55]. Body weight analysis did not find weight loss in the 4OI treatment group (Fig. 5I). In general, these measurements indicate that systemic 4OI therapy is effective within an acceptable range of toxicity.

## Discussion

**Fig. 6:**
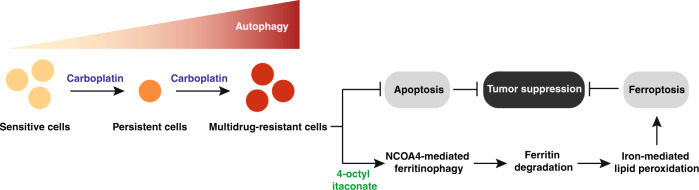
Schematic summary of targeting autophagy for RB therapy. Persistent cells are a small subgroup of cancer cells that remain viable under carboplatin treatment, leading to multidrug resistance by increasing autophagy activity. Up-regulated autophagy inhibits caspase-mediated apoptosis, but increases the sensitivity of drug-resistant RB cells to ferroptosis. In particular, 4-octyl itaconate activates NCOA4-mediated ferritinophagy, leading to ferritin degradation and subsequent free iron accumulation, and finally oxidative damage through the iron-mediated ROS production via Fenton reaction.

The diagnosis and treatment of cancer is difficult, but rare cancers are especially challenging for patients, their caregivers, and even clinicians. The development of chemotherapy resistance, including primary, adaptive, and acquired resistance, remains a serious obstacle for treating RB patients [4]. In this study, we demonstrate that itaconate-induced ferritinophagy drives ferroptotic death to eliminate drug-resistant human RB cells (Fig. 6). This strategy is different from the current mainstream anticancer strategies in that it acts by inhibiting the autophagy-mediated survival pathway, thereby providing emerging opportunities for treatment of rare cancers.

MDR refers to the reaction of cancer cells that are resistant to multiple chemotherapeutic drugs simultaneously after being exposed to one chemotherapeutic drug [56]. We observed that carboplatin-resistant RB cells are also resistant to other commonly used anticancer drugs in RB therapy, including etoposide and vincristine. A classical explanation for the formation of MDR is that abnormal changes in the drug pumps on the cell membrane affect the accumulation of drugs in the cell [56]. In addition to this well-recognized drug excretion mechanism, our data reveal that increased autophagy is responsible for MDR in RB cells. It seems that many chemotherapeutic drugs are apoptosis inducers [57], and autophagy can degrade the apoptotic effector caspases [58, 59]. Thus, it is not surprising that inhibiting autophagy restores the activity of drug-resistant RB cells to carboplatin, etoposide, and vincristine.

What do the current results mean for therapeutic targeting of autophagy in cancer? Although autophagy inhibition has shown some promising activities in tumor treatment, clinical trials based on the combination of chloroquine/hydroxychloroquine and chemotherapy have not yet achieved major breakthroughs [60]. The exact reason for this situation is unclear, but it may be related to the off-target effects of chloroquine/hydroxychloroquine [61] or the side effects of inhibiting autophagy in normal cells or tissues [20]. For example, autophagy plays a key role in many aspects of the immune system, including the development and function of T lymphocytes [62]. Inhibition of autophagy may impair the function of CD8^+^ T cells in anti-tumor immunity [63]. In addition to the continuous development of new and specific drugs to selectively inhibit autophagy in cancer cells, our current research proposes a different anticancer strategy that uses the increased autophagy activity in drug-resistant cells to trigger autophagy-dependent cell death. The susceptibility of drug-resistant cells to this selective autophagy induction support further relevant clinical trials to inducing autophagy-mediated ferroptosis through itaconate derivative.

In tumor therapy, non-apoptotic cell death pathways can be used to inhibit apoptosis-resistant cells [64]. Due to different signals and mediators of cell death modalities, it is necessary to carefully evaluate whether the key components of the cell death machinery are intact in different cancers [65]. Our analysis reveals that the chemical targets (SLC7A11 and GPX4) of ferroptosis inducers are overexpressed in carboplatin-resistant RB cells. However, the key regulators of necroptosis (RIPK1 and MLKL) are not different or are not expressed (RIPK3) when comparing the parental and carboplatin-resistant RB cells. Consequently, a variety of ferroptosis inducers, rather than the necroptosis inducer CCT137690 [34], effectively eliminate drug-resistant RB cells. Therefore, despite the differences in ferroptosis inducers, their numerous similarities make them valuable tools for future research and treatment testing.

We also demonstrate that targeting ferroptosis is more effective in drug-resistant RB cells compared to parental cells. This difference exists because ferroptosis is a type of autophagy-dependent cell death [66], and drug-resistant RB cells have a higher level of autophagy. Ferroptosis was originally described as a type of cell death that does not rely on autophagy machinery [35]. Recent studies challenge this view and prove that ferroptosis activators increase autophagy flux, and autophagy-deficient cells are resistant to ferroptotic damage or death [43]. These updated findings may provide a theoretical basis for the development of ferroptosis-targeting strategies to suppress tumors with high levels of autophagy [67]. Mechanistically, autophagy promotes ferroptosis by selectively degrading anti-ferroptotic proteins (e.g., ferritin [44], GPX4 [68, 69], ARNTL [aryl hydrocarbon receptor nuclear translocator like] [70], and SLC40A1 [solute carrier family 40 member 1] [71]) or organelles (e.g., lipid droplets [72]), thereby promoting iron accumulation and lipid peroxidation. In this study, we confirm that NCOA4-mediated ferritinophagy, a type of selective autophagy for the degradation of ferritin [45], is important for killing drug-resistant RB cells through ferroptosis. Because tumor heterogeneity affects the sensitivity of autophagy-dependent ferroptosis [71], it is necessary to evaluate whether this targeting strategy is applicable to other types of cancer.

We determined that 4OI is a strong ferroptosis inducer by activating ferritinophagy. At present, the classic ferroptosis inducers are synthetic small molecule compounds. However, due to metabolic instability, most of them have limited anticancer activity when used in vivo [33]. 4OI is a cell-permeable itaconate derivative, which acts as an immunometabolite to diminish inflammation in innate immunity [49]. Tumor-associated macrophages also produce itaconate to shape the tumor microenvironment [73]. Consistent with the finding that excessive exogenous itaconate is toxic to pancreatic cancer cells [48], we demonstrate that 4OI triggers ferritinophagy-dependent ferroptosis, thereby inhibiting the growth of RB cells. In addition to effectiveness, our animal studies also confirm that the administration of 4OI is generally safe for mice. Nevertheless, the long-term impact of 4OI as well as its upstream regulator ACOD1 (aconitate decarboxylase 1) on immunity, metabolism, and cell death needs further evaluation [74].

In conclusion, we reveal an autophagy-dependent cell death mechanism that favors the elimination of multidrug-resistant RB cells, thus offering therapeutic targets for interfering with acquired resistance to the current fist-line chemotherapy. The demonstration that 4OI has pro-autophagic and pro-ferroptotic activity may favor the development of a new metabolite strategy for tumor treatment.

## Materials and methods

### Reagents

Erastin (S7242), RSL3 (S8155), liproxstatin-1 (S7699), Z-VAD-FMK (S7023), FIN56 (S8254), necrosulfonamide (S8251), CCT137690 (S2744), oxaliplatin (S1224), BMS-345541 (S8044), carboplatin (S1215), etoposide (S1225), vincristine (S9555), deferoxamine (S5685), chloroquine (S6999), and 4OI (S5929) were purchased from Selleck Chemicals. The antibodies to SQSTM1 (5114), ATG5 (2630), MLKL (14993), NCOA4 (66849), FTH1 (3998), ATG7 (2631), SLC7A11 (12691), and ACTB (3700) were purchased from Cell Signaling Technology. The antibodies to MAP1LC3B (NB100-2220), RIPK1 (NBP1-77077), and RIPK3 (NBP1-77299) were purchased from NOVUS. The antibody to GPX4 (ab125066) was purchased from Abcam.

### Cell culture and treatment

Y79 (HTB-18) and WERI-Rb-1 (HTB-169) were obtained from the American Type Culture Collection. The carboplatin-resistant Y79-CR cell line was established by exposing Y79 cells to carboplatin through the limiting dilution method [27]. Cells were cultured in RPMI 1640 (Thermo Fisher Scientific, 11875119) supplemented with 10% heat-inactivated fetal bovine serum (Millipore, TMS-013-B) and 1% penicillin and streptomycin (Thermo Fisher Scientific, 15070-063) at 37 °C, 95% humidity, and 5% CO_2_. All cells were mycoplasma free and authenticated using short tandem repeat DNA profiling analysis. Dimethyl sulfoxide (DMSO; VWR International, IC0219605525) was used to prepare the stock solution of drugs. The final concentration of DMSO in the drug working solution in the cells was <0.01%. DMSO of 0.01% was used as a vehicle control in all cell culture assays.

### Animal models

Mice were housed on a regular 12-h light and dark cycle (7:00-19:00 light period; room temperature: 20–25 °C; relative humidity: 40–60%). Food and water were available *ad libitum*. Experiments were carried out under pathogen-free conditions and the health status of mouse lines was routinely checked by veterinary staff. Experiments were carried out with randomly chosen littermates of the same sex and matched by age and body weight. We conducted all animal care and experiments in accordance with the Association for Assessment and Accreditation of Laboratory Animal Care guidelines (http://www.aaalac.org) and with approval from our institutional animal care and use committee.

To generate murine subcutaneous tumors, 2 × 10^6^ Y79 or Y79-CR cells mixed with phosphate buffered saline (pH 7.4; Thermo Fisher Scientific, 10010023) to a final volume 50 µl of were subcutaneously injected into the flanks of recipient female nude mice (4 weeks old; 20–22 g of body weight) using a 27-gauge needle for 7 days, and then treated (intraperitoneally) with 4OI (50 mg/kg) or vehicles for two weeks (once every other day). Tumors were measured weekly, and volumes were calculated using the formula length × width^2^ × π/6. On day 21 after 4OI or vehicles treatment, the animals were sacrificed, and serum and tumor tissue samples were collected for further measurement; none were excluded from analysis at the time of harvest. All treatments were performed by technicians who were not blinded to the experiment but were not involved in sample measurement.

### Western blot analysis

Cells were lysed three times with cell lysis buffer (Cell Signaling Technology, 9803) containing protease inhibitor (Thermo Fisher Scientific, 78429) on ice for 10 min. Protein was quantified using the bicinchoninic acid assay (Thermo Fisher Scientific, 23225) and 20–30 μg of each sample was resolved on 4–12% Criterion XT Bis-Tris gels (Bio-Rad, 3450124) in XT MES running buffer (Bio-Rad, 1610789) and transferred to polyvinylidene difluoride membranes (Bio-Rad, 1620233) using the Trans-Blot Turbo Transfer Pack and System. Membranes were blocked with TBST buffer containing 5% skim milk for 1 h and incubated overnight at 4 °C with various primary antibodies (1:1000). Following three washes in TBST, membranes were incubated with goat anti-rabbit or anti-mouse IgG HRP secondary antibody (1:1000; Cell Signaling Technology, 7074 or 7076) at room temperature for 1 h and washed. Chemiluminescence substrate was applied using SuperSignal West Pico Chemiluminescent Substrate (Thermo Fisher Scientific, 34580) or SuperSignal West Femto Maximum Sensitivity Substrate (Thermo Fisher Scientific, 34095), and signals were analyzed using the ChemiDoc Touch Imaging System (Bio-Rad) or x-ray films. The relative density was displayed at the bottom of the band, and the control was set to 1. The uncropped original western blots was shown in Supplemental Material.

### Cell viability and death assays

Cells were seeded into 96-well plates and incubated with the indicated treatments. Subsequently, 100 μl of fresh medium was added to cells containing 10 μl of Cell Counting Kit-8 solutions (Dojindo Laboratories, CK04) and incubated for 1–1.5 h (37 °C, 5% CO_2_). Absorbance at 450 nm was measured using a microplate reader (Tecan). The level of cell death was assayed using a LIVE/DEAD cell viability/cytotoxicity assay kit (Thermo Fisher Scientific, L3224) according to the manufacturer’s protocol. The viability or death without drug or chemical treatment or in the presence of control shRNA was set to 100% or 0%, respectively, and other values were normalized.

### RNAi

The predesigned human *ATG5* shRNA-1 (TRCN0000330394), *ATG5* shRNA-2 (TRCN0000151474), *ATG7* shRNA-1 (TRCN0000007584), *ATG7* shRNA-2 (TRCN0000364479), *ACSL4* shRNA-1 (TRCN0000045541), *ACSL4* shRNA-2 (TRCN0000045539), *NCOA4* shRNA (TRCN0000236186), and control empty shRNA (pLKO.1) in a lentiviral format were obtained from Sigma-Aldrich. We seeded 1 × 10^5^ cells in each well of a 12-well plate in 500 μl of complete medium and transduced them by lentiviral vectors at a multiplicity of infection of 10:1. Transduction was carried out in the presence of polybrene (8 μg/ml; Thermo Fisher Scientific, TR1003G) in an antibiotic-free medium. After recovering with complete culture medium, puromycin (Thermo Fisher Scientific, A1113802, 5 μg/ml) was used for the selection of transduced cells. The efficiency of RNAi was checked by western blot analysis of target proteins.

### Autophagy analysis

The GFP-LC3-RFP-LC3ΔG probe was a gift from Dr. Noboru Mizushima (Addgene, 84572), which is a simple and quantitative method to evaluate autophagic flux [30]. The cysteine protease ATG4 cleaves this probe into a degradable part (GFP-LC3, which can be delivered to the lysosome via autophagy) and a stable part (RFP-LC3ΔG, which remains in the cytosol) upon autophagic stimulus [30]. Thus, a decrease of the GFP:RFP ratio indicates the occurrence of autophagic flux [30]. In brief, the indicated RB cells (5000 cells/well) expressing GFP-LC3-RFP-LC3ΔG in a black 96-well plate with a clear bottom (Corning, 3904) were treated with carboplatin, etoposide, vincristine, or 4OI at the indicated concentrations for 6 h, and the signal of GFP and RFP was analyzed using a microplate reader (Tecan). In the absence or presence of chloroquine, the turnover of MAP1LC3B and SQSTM1 was assayed in the indicated RB cells by western blot.

Transmission electron microscopy analysis of autophagic vacuoles was performed as previously described [75]. In brief, cells were fixed with 2% paraformaldehyde and 2% glutaraldehyde in 0.1 mol/L phosphate buffer (pH 7.4), followed by postfixation for 6 h in 1% OsO_4_. After dehydration with graded alcohols, the sample was embedded in epoxy resin (Sigma-Aldrich, 45359). The cut-thin sample (70 nm) was mounted on a copper mesh and post-stained with 2% uranyl acetate and 1% lead citrate, dried, and analyzed with a transmission electron microscope (JEOL).

### Biochemical assay

Commercially available enzyme-linked immunosorbent assay (ELISA) kits were used to measure the concentrations or activity of CASP3 (Cell Signaling Technology, 5723), and HMGB1 (Shino-Test Corporation, ST51011) in the indicated samples according to the manufacturers’ instructions. Measurement of GPT/ALT and BUN in the serum was performed using a Catalyst Dx Chemistry Analyzer (IDEXX).

The lipid ROS was measured using a BODIPY 581/591 C11 indicator (Thermo Fisher Scientific, D3861) according to the manufacturer’s protocol. Oxidation of the polyunsaturated butadienyl portion of the dye results in a shift of the fluorescence emission peak from ∼590 nm to ∼510 nm. The rate of cellular lipid oxidation was assessed by monitoring the changes in green fluorescence of untreated or treated cells with 5 μM BODIPY 581/591 C11 for 30 min in a black 96-well plate (Corning, 3904) using a fluorescence plate reader (Tecan). The fluorescence signal was normalized to the untreated group and set to 100%.

The relative MDA concentration in cell lysates was assessed using a Lipid Peroxidation Assay Kit (Abcam, ab118970) according to the manufacturer’s instructions. Briefly, the MDA in the sample reacted with thiobarbituric acid (TBA) to generate a MDA-TBA adduct. The MDA-TBA adduct were quantified colorimetrically (OD = 532 nm) or fluorometrically (Ex/Em = 532/553 nm).

The relative Fe^2+^ concentration in cells was assessed using an Iron Assay Kit (Sigma-Aldrich, MAK025). Briefly, cells or tissues were homogenized in 4–10 volumes of iron assay buffer, and the samples were centrifuged at 16,000 × *g* for 10 min to remove insoluble materials, followed by collection of the supernatants. To measure ferrous iron, we added 50 µL samples to sample wells in a 96-well plate and brought the volume to 100 µL per well with 5 µL of assay buffer. After incubation of the reaction at 37 °C for 30 min, the absorbance at 593 nm was measured using a microplate reader. The relative level of Fe^2+^ in all groups was calculated and normalized to protein concentration.

### Calcium flux assay

Fluo-8 (Abcam, ab112129) is a novel green calcium indicator to monitor calcium concentration and flux in cells. Briefly, the level of cytosolic calcium was assessed by monitoring the changes in green fluorescence of untreated or treated cells with 5 μM Fluo-8 for 30 min in a black 96-well plate (Corning, 3904) using a fluorescence plate reader (Tecan) at Ex/Em 490/525 nm. The fluorescence signal was normalized to the untreated group and set to 100%.

### Mitochondrial membrane potential assay

Mitochondrial membrane potential changes in cells were assessed using the membrane-permeant JC-1 dye (Thermo Fisher Scientific, M34152) according to the manufacturer’s protocol. JC-1 is a lipophilic cationic dye that selectively enters mitochondria and reversibly changes from green (Ex/Em 514/529 nm) to red (Ex/Em 514/590 nm) with increasing membrane potential. The red to green ratio is decreased as the membrane potential decreases. Briefly, indicated cells were incubated with 2.5 μM JC-1 in a black 96-well plate (Corning, 3904) at 37°C for 15 min. The fluorescence signals were analyzed on a fluorescent microplate reader (Tecan). The red/green fluorescence ratio was calculated. The untreated group was set to 1.

### Statistical analysis

GraphPad Prism 8.4.3 was used to collect and analyze data. A one-way or two-way analysis of variance (ANOVA) with Tukey’s multiple comparisons test was used for comparisons among the different groups. A *P* value of <0.05 was considered statistically significant. We did not exclude samples or animals. No statistical methods were used to predetermine sample sizes in animal studies, but our sample sizes are like those generally employed in the field. The investigators were not blinded to allocation during experiments and outcome assessment.

## Supplementary information

Checklist

Western blot - raw data

## Data Availability

The published article includes all data generated or analyzed during this study.
