# Supplementary figures and images for: Induction of autophagy-dependent ferroptosis to eliminate drug-tolerant human retinoblastoma cells
Source: Cell Death Dis. 2022 Jun 2;13(6):521. doi: 10.1038/s41419-022-04974-8 (PMC9163041; doi:10.1038/s41419-022-04974-8)

Fig.1D

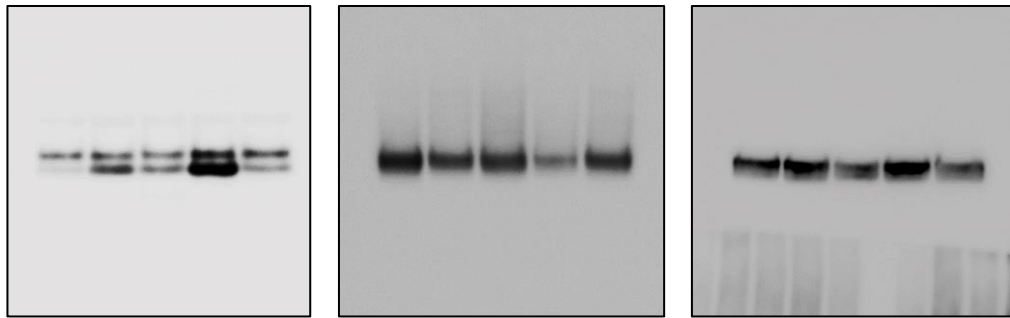

Fig.1G

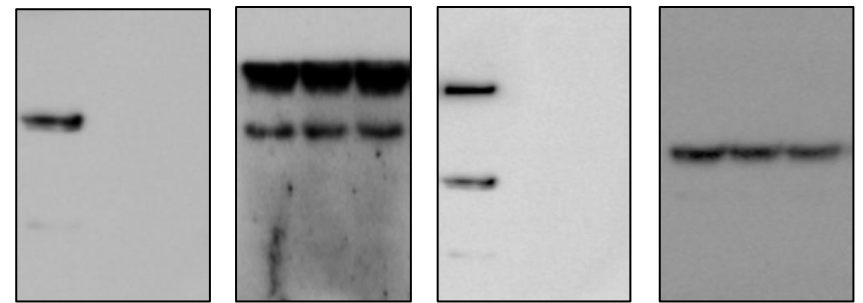

Fig.2C

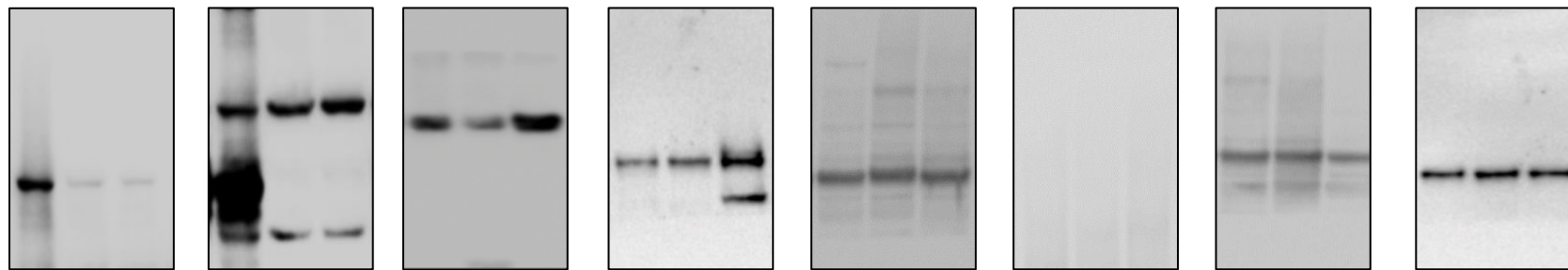

Fig.3A

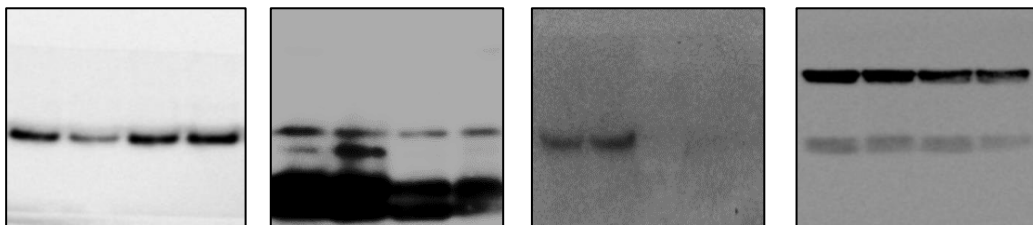

Fig.3C

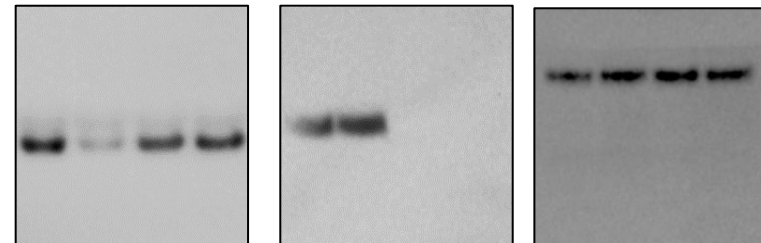

Fig.4E

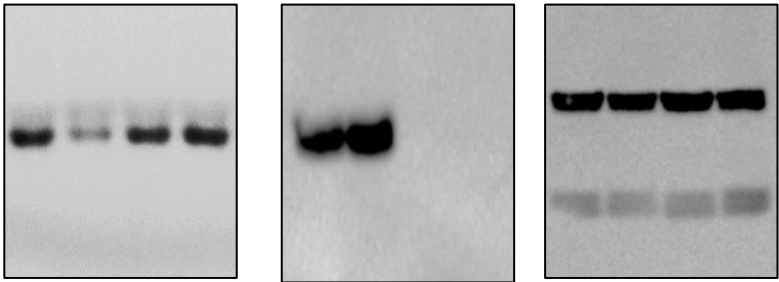

Fig.4K

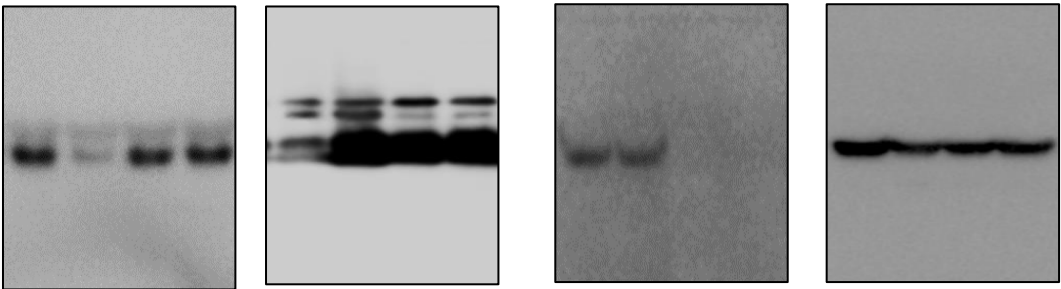

Fig.5B

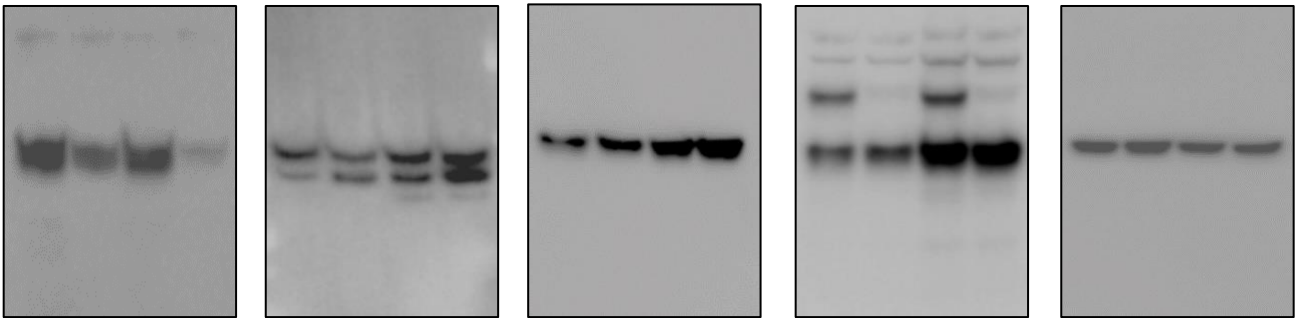

Supplement: Supplementary file 2 — Western blot - raw data [file 41419_2022_4974_MOESM2_ESM.pdf]
